# Osteocytic Sclerostin Expression as an Indicator of Altered Bone Turnover

**DOI:** 10.3390/nu15030598

**Published:** 2023-01-23

**Authors:** Yentl Huybrechts, Pieter Evenepoel, Mathias Haarhaus, Etienne Cavalier, Geert Dams, Wim Van Hul, Patrick C. D’Haese, Anja Verhulst

**Affiliations:** 1 Laboratory of Pathophysiology, University of Antwerp, 2610 Antwerp, Belgium; 2 Center of Medical Genetics, University of Antwerp, 2650 Antwerp, Belgium; 3 Laboratory of Nephrology, Department of Immunology and Microbiology, KU Leuven, 3000 Leuven, Belgium; 4 Division of Renal Medicine, Department of Clinical Science, Intervention and Technology, Karolinska Institutet, Campus Flemingsberg, 141 52 Stockholm, Sweden; 5 Diaverum AB, 215 37 Malmö, Sweden; 6 Department of Clinical Chemistry, CHU de Liège, Université de Liège, 4000 Liège, Belgium

**Keywords:** chronic kidney disease (CKD), renal osteodystrophy (ROD), parathyroidectomy (PTX), bone turnover, osteocyte, sclerostin, parathyroid hormone (PTH)

## Abstract

Renal osteodystrophy (ROD) is a complex and serious complication of chronic kidney disease (CKD), a major global health problem caused by loss of renal function. Currently, the gold standard to accurately diagnose ROD is based on quantitative histomorphometric analysis of trabecular bone. Although this analysis encompasses the evaluation of osteoblast and osteoclast number/activity, tfigurehe interest in osteocytes remains almost nihil. Nevertheless, this cell type is evidenced to perform a key role in bone turnover, particularly through its production of various bone proteins, such as sclerostin. In this study, we aim to investigate, in the context of ROD, to which extent an association exists between bone turnover and the abundance of osteocytes and osteocytic sclerostin expression in both the trabecular and cortical bone compartments. Additionally, the effect of parathyroid hormone (PTH) on bone sclerostin expression was examined in parathyroidectomized rats. Our results indicate that PTH exerts a direct inhibitory function on sclerostin, which in turn negatively affects bone turnover and mineralization. Moreover, this study emphasizes the functional differences between cortical and trabecular bone, as the number of (sclerostin-positive) osteocytes is dependent on the respective bone compartment. Finally, we evaluated the potential of sclerostin as a marker for CKD and found that the diagnostic performance of circulating sclerostin is limited and that changes in skeletal sclerostin expression occur more rapidly and more pronounced. The inclusion of osteocytic sclerostin expression and cortical bone analysis could be relevant when performing bone histomorphometric analysis for diagnostic purposes and to unravel pathological mechanisms of bone disease.

## 1. Introduction

Bone remodeling is initiated by osteocytes, the most abundant bone cell type and the so-called mechanosensors of bone [1]. Osteocytes are embedded in the mineralized bone matrix and can react to microdamage and mechanical loading, resulting in the recruitment of osteoclast precursors and the stimulation of bone formation by activating the canonical Wnt/β-catenin signaling pathway [2,3]. Sclerostin, encoded by the *SOST* gene, is an extracellular inhibitor of this pathway through binding to a Frizzled receptor and the low-density lipoprotein receptor-related protein (LRP) 5/6 co-receptor [4,5]. Sclerostin is predominantly expressed by osteocytes, an often-overlooked bone cell type during histomorphometric bone analysis. In contrast to the osteoblast and osteoclast, the association between the number and activity of the osteocyte with bone turnover has not been thoroughly studied, despite the fact that sclerostin, amongst several other osteocyte-derived factors performing a role in bone metabolism/mineral homeostasis, has received particular interest within the chronic kidney disease-mineral bone disorder (CKD-MBD) research community [4,6,7].

CKD is characterized by a progressive loss of renal function, which may eventually lead to a heterogenous group of bone disorders, collectively known as renal osteodystrophy (ROD) [8,9]. These bone pathologies can be classified based on three key parameters: bone turnover, mineralization and volume (TMV) [10]. To date, quantitative histomorphometric analysis of trabecular bone is considered the gold standard for diagnosis [11]. However, bone biopsy is invasive and analysis is labor-intensive and performed in only a limited number of laboratories, making an early diagnosis almost impossible [12,13]. Therefore, the identification of valid histological and/or biochemical biomarkers would be much less burden for the patient and the researchers and is currently subject to extensive research. In this regard, it is noteworthy that KDIGO (Kidney Disease: Improving Global Outcomes) recommends measuring parathyroid hormone (PTH) and bone alkaline phosphatase (BALP) levels to monitor disease progression and pharmacological treatment [14].

It is known since long that PTH performs a crucial role in ROD [15,16]. More specifically, PTH is a highly important regulator of calcium and phosphorus homeostasis, which is maintained through a complex relationship between the bones, intestine, kidneys and parathyroid glands and failure of one of these organs will have a deleterious effect on the others [17,18]. Although PTH is used as an indicator of bone turnover in CKD, its diagnostic performance as a biomarker is limited [19,20,21,22]. More specifically, PTH levels are highly dependent on external factors, such as age and kidney function, and skeletal hyposensitivity to PTH in CKD was previously reported. Furthermore, the high biological variability in combination with the lack of standardized assays is detrimental to accurately diagnose ROD [21,23]. Despite BALP being independent of renal function, studies show that it also cannot accurately distinguish between low, normal and high bone turnover in CKD-ROD [14,24]. Hence, there has been a continuous search to alternative bone biomarkers, which amongst others led to the identification of sclerostin as a potential candidate. As mentioned earlier, sclerostin is an inhibitor of the WNT signaling pathway, allowing it to directly reflect changes in bone metabolism. Since other markers often work differently, implementing sclerostin—alone or in combination with other biomarkers—can benefit the diagnostic landscape. It has already been extensively described that serum levels of sclerostin increase as CKD progresses [6,7,25]. However, research on the involvement of sclerostin in ROD and bone turnover in particular is rather limited and data concerning its expression in different bone compartments, i.e., trabecular versus cortical bone.

To facilitate progress in the field of biomarkers, it may be useful to further elucidate the complex pathology of ROD. During the last years, interest has focused on the interaction between PTH and sclerostin and their effects on different bone processes [14,21,24,26,27]. Hence, the aim of this study is to further unravel the PTH-sclerostin interaction and to evaluate the diagnostic potential of sclerostin as a biomarker by investigating its role in bone metabolism, turnover and mineralization.

## 2. Materials and Methods

### 2.1. Parathyroidectomized (PTX) Rat Model

Twenty male Wister rats with normal renal function were randomly divided into two study groups: rats that underwent parathyroidectomy (PTX group, *n* = 10) and a control group of sham-operated rats (non-PTX group, *n* = 10). The animals had free access to fresh water and were fed a standard diet (1% calcium, 0.70% phosphorus, 1500 IU/kg vitamin D_3_ (labeled values; V1534-300; Ssniff Spezialdiäten GmbH, Soest, Germany)).

Throughout the study period, blood samples were taken every 2 weeks from the tail vein. Phosphate, calcium, magnesium, fibroblast growth factor 23 and vitamin D were measured using standard techniques [28]. Serum intact PTH (iPTH) and sclerostin levels were assessed using enzyme-linked immuno sorbent assays (ELISA, Immunotopics and Cloud-Clone Corp. resp., Katy, TX, USA), according to manufacturer’s instructions. After 12 weeks, rats were euthanized and the left tibia was isolated for quantitative bone histomorphometric analysis. In order to allow the assessment of dynamic bone parameters rats were labeled intravenously with tetracycline (30 mg/kg) and demeclocycline (25 mg/kg) 7 and 3 days before euthanization, as we described previously [28]. This procedure was approved by the Ethics Committees of the University of Antwerp (Belgium).

### 2.2. Patient Cohort

The cohort consisted of 30 patients (19 males), which were included after informed consent, in collaborative bone biopsy-based studies with the University Hospital Leuven (Belgium) and the Karolinska University Hospital in Stockholm (Sweden). Patients underwent a transiliac bone biopsy after double tetracycline labeling. The majority of patients were classified as kidney transplant (Tx) patients (*n* = 20), whereas the other patients have a CKD5 status (*n* = 10). A CKD5 status was given to patients with renal failure at the time of biopsy (eGFR < 15 mL/min/1.73 m^2^, official criteria [29]). All other patients were labeled as “transplant patients” as they received a kidney transplant and had an eGFR > 15 mL/min/1.73 m^2^. Patients were allocated to one of three groups, based on their bone formation rate (BFR, expressed in µm^2^/mm^2^/day): low (BFR < 97), normal (97 ≤ BFR ≤ 613) or high (BFR > 613) bone turnover. This allocation was based on criteria used in our lab for the classification of ROD and as published by Behets et al. [30]. Blood samples were taken at the time of bone biopsy and analyzed at the respective institutions. All samples were analyzed at the University of Antwerp to ensure standardization. All patients were older than 18 years of age and ethical approval was granted by the regional ethics committees in Leuven and Stockholm.

### 2.3. Serum Biochemistry

Creatinine, calcium, phosphate, 25-OH Vitamin D and total alkaline phosphatase (ALP) were determined in serum samples of all patients using routine methodologies. Serum levels of iPTH (BIO intact) were determined as described elsewhere [31]. Bone alkaline phosphatase (BALP, IDS-iSYS), *N*-terminal propeptide of type I procollagen (PINP, IDS-iSYS), tartrate-resistant acid phosphatase 5b (TRAP5b, IDS-iSYS) and sclerostin (Elisa TECO medical) were determined according to manufacturer’s instructions.

### 2.4. Bone Histomorphometric Analysis

Bone tissue of human patients and rats was dehydrated by passing sequentially through several alcohol solutions and subsequently embedded in methyl methacrylate. In line with the conventional procedures for bone histomorphometric classification of ROD, the static and dynamic parameters were evaluated in trabecular bone tissue, as described elsewhere [30,32]. In brief, 5 µm thick sections were Goldner stained in order to assess the static bone parameters, while dynamic parameters were determined using 10 µm unstained fluorescent sections (tetracycline labeled). All parameters, their units and abbreviations are specified in Supplementary Table S1. Histomorphometric analysis of all samples was performed by microscopically scanning the sections using a custom developed software program for image analysis (AxioVision Release 4.5).

### 2.5. Osteocyte Quantification

To quantify the osteocytes in human and rat skeletal tissue, mineralized bone and osteoid were delineated on Goldner stained sections, followed by manual counting of the cells. Thereby, a distinction was made between the different bone compartments (i.e., cortical and trabecular bone) and between empty lacunae and osteocytes with a visible cell nucleus, in order to avoid the inclusion of artefacts. Whereas the human biopsies were entirely scanned, the analyses of the rat samples were limited to six adjacent fields, due to the abundance of osteocytes and to ensure standardization of the evaluated regions. Each time, half a field separation was kept between the field to be measured and the growth plate. All results are reported as the number of cells per cortical or trabecular bone area (in mm^2^).

### 2.6. Quantification of Sclerostin-Positive Osteocytes

Immunohistochemical staining was performed to determine the osteocytic sclerostin expression in all samples. First, 5 µm thick bone sections of rats/patients were de-acrylated, rehydrated and decalficied. Next, several blocking steps were included to increase specificity, i.e., endogenous peroxidase (3% hydrogen peroxide in methanol for 15 min), avidin (1/200 in phosphate-buffered saline (PBS) for 20 min), biotin (0.5 mg/mL in PBS for 20 min) and normal goat serum (1/5 in PBS for 20 min). The sections were incubated overnight with a primary rabbit-derived polyclonal anti-sclerostin antibody (1/50 in PBS, Santa Cruz Biotechnology, Dallas, TX, USA). A biotinylated secondary goat anti-rabbit antibody (Vector Laboratories, Newark, CA, USA) was used, followed by adding avidin/biotinylated peroxidase complexes (Vectastain ABC kit, Vector Laboratories, Newark, CA, USA) and 3-amino-9-ethylcarbazole (Sigma-Aldrich, St. Louis, MI, USA) as a chromogenic substrate. In between steps, rinsing was conducted alternately with distilled water or PBS. For all experiments, bone sections without incubation with the primary antibody were used as a negative control. The quantification of sclerostin-positive osteocytes per bone area was conducted by delineating the bone tissue and manually counting the positive cells, using a custom developed program on image analysis software (AxioVision Release 4.5). Again, the trabecular and cortical compartments of human biopsies were entirely scanned, whereas for the rat biopsies, six adjacent fields per compartment/section were examined. All results are reported as number of cells per cortical or trabecular bone area (in mm^2^).

### 2.7. Statistical Analysis

Statistical analysis was performed using IBM SPSS Statistics 27. Non-parametric testing was applied for all analyses, given the sample size (*n* = 10/group), and data is expressed as median and interquartile range (IQR). To compare the different study groups, a Kruskall–Wallis Test and subsequent Mann–Whitney U Test were performed. Bonferroni correction was conducted to avoid statistical errors due to multiple testing. Associations between the cell counts and histomorphometric or biochemical parameters were established using Spearman Rank Correlations. A *p*-value ≤ 0.05 was considered statistically significant.

## 3. Results

### 3.1. PTH Exerts a Direct Inhibitory Effect on Bone Sclerostin Expression in PTX Rats

#### 3.1.1. Characteristics of (Non-)PTX Rats

**Figure 1:**
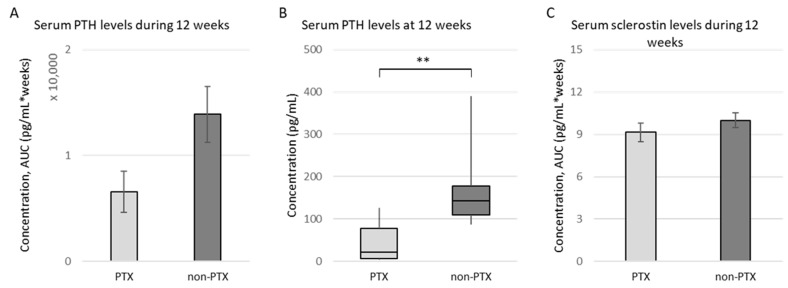
Serum iPTH and sclerostin levels. Serum iPTH and sclerostin levels were measured in PTX rats (*n* = 10) and non-PTX rats (*n* = 9) using specific enzyme-linked immunosorbent assays (ELISA). (**A**) Total exposure of the bone to iPTH during the 12 weeks of the study (measured as area under the curve (AUC), pg/mL * weeks); (**B**) Serum iPTH levels at 12 weeks (shown as median ± IQR); (**C**) Total exposure of the bone to sclerosin during the 12 weeks of the study (measured as AUC, pg/mL * weeks). ** *p* ≤ 0.01.

One animal of the non-PTX group died shortly after surgery. Although the PTX did not lead to a complete inhibition of iPTH production, most probably due to incomplete removal of the parathyroid gland inherent to the high complexity of this procedure in rats, the total exposure of the bone to iPTH during the 12 weeks of the study was clearly reduced in PTX rats (Figure 1A). Eventually, a significant reduction in serum iPTH levels was observed 12 weeks after surgery (average values (± SD) of 176.62 ± 99.39 pg/mL for non-PTX versus 45.24 ± 46.97 pg/mL for PTX rats; Figure 1B). No significant differences in phosphate, calcium, magnesium, fibroblast growth factor 23 or vitamin D were seen at 12 weeks. With regard to histomorphometric parameters determined in trabecular bone tissue of both groups, no significant differences could be observed between PTX and non-PTX animals (Table S2). Regarding serum sclerostin levels, no difference between non-PTX and PTX rats could be observed during the study (Figure 1C).

#### 3.1.2. Quantification of (Sclerostin-Positive) Osteocytes in Bone of (Non-)PTX Rats

**Figure 2:**
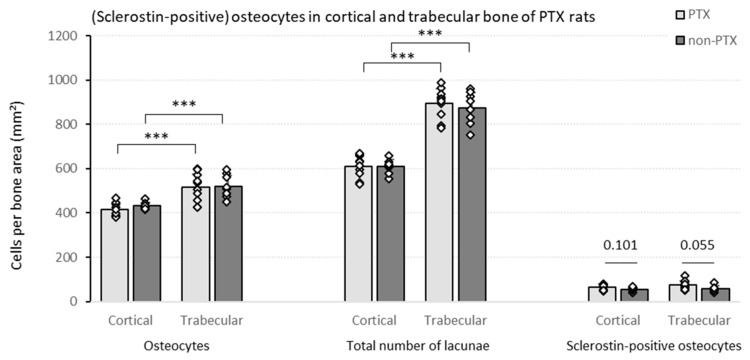
Cell counts in (non-)PTX RATS. Different cell counts (i.e., osteocytes, total number of lacunae and sclerostin-positive osteocytes) were determined in the cortical and trabecular bone of PTX rats (*n* = 10) and non-PTX rats (*n* = 9). Data shown as mean ± SD. *** *p* ≤ 0.001.

The histological quantification of the number of osteocytes shows that there are no significant differences between the PTX and the non-PTX group in terms of osteocyte count (Figure 2). Comparing both bone compartments, a significantly higher number of osteocytes per bone area was observed in trabecular versus cortical bone in both rat groups. A similar, even somewhat more pronounced, pattern was seen when the total number of lacunae—i.e., sum of cell nuclei containing lacunae and empty lacunae—was considered. Interestingly, a nominally higher sclerostin positivity was noticed in the PTX group compared to the non-PTX rats. In the trabecular bone, this difference reached borderline statistical significance (*p* = 0.055), hence pointing towards a possible direct effect of iPTH on osseous sclerostin expression.

To put the findings mentioned above in perspective with regard to bone metabolism, correlations were calculated with both static and dynamic trabecular bone histomorphometric parameters (Table 1). In the non-PTX group, the number of osteocytes per bone area correlated negatively with bone area (B.Ar) and osteoblast perimeter (Ob.Pm.O). Similar results were observed when the total number of lacunae was taken into account (data not shown).

The number of sclerostin-positive osteocytes per bone area was associated with impaired bone mineralization, as indicated by the significant negative correlation with the adjusted apposition rate (Aj.AR) and positive association with the mineralization lag time (Mlt), specifically in PTX rats (Table 1).

#### 3.1.3. Serum iPTH Is Inversely Correlated with Sclerostin Expression in Trabecular Bone

Given the aforementioned results showing an upregulation of sclerostin in PTX versus non-PTX rats, one might reasonably expect bone sclerostin expression to negatively correlate with serum iPTH levels. It was observed that there was a negative association between serum iPTH and bone sclerostin expression, in trabecular bone of the PTX group only (Table 2).

### 3.2. Sclerostin Expression, Number of Osteocytes and Bone Histomorphometry in Patients with Different Degrees of Bone Turnover

#### 3.2.1. Demographics and Specific Characteristics of the Cohort

Thirty patients (CKD5 or transplant) with different degrees of bone turnover, were divided into three groups (*n* = 10/group): low, normal and high bone turnover according to the bone formation rate (BFR). As demonstrated in Table 3, the increasing bone turnover is further evidenced by a significant increase in the amount of osteoid (O.Ar, O.pm and O.Wi), number of osteoblasts per total perimeter (Ob.Pm.T) and Aj.AR and decreasing Mlt. No differences could be observed between groups with regard to age and serum parameters reflecting mineral biochemistry, e.g., calcium and phosphate. In contrast, serum levels of iPTH, ALP, BALP, PINP and TRAP5b significantly increased with increasing bone turnover. Remarkably, serum sclerostin levels did not differ between the different bone turnover groups. In this context, it is worth to mention that kidney function, measured by eGFR and creatinine levels, is increased in the high bone turnover group (Table 3). Hence, this might have compensated for the low sclerostin levels that would reasonably be expected given the high iPTH levels in the high bone turnover group.

#### 3.2.2. Quantification of (Sclerostin-Positive) Osteocytes in Patients with Varying Degrees of Bone Turnover

**Figure 3:**
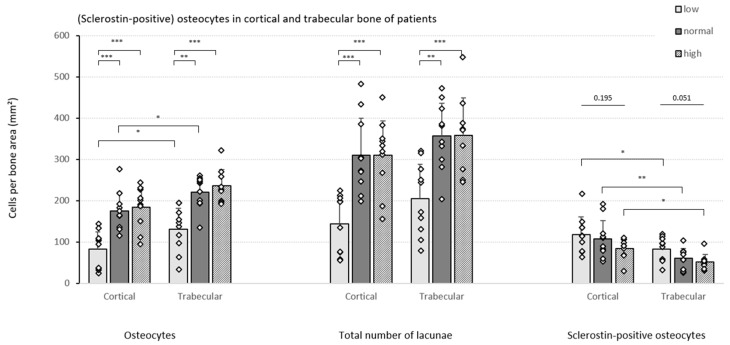
Cell counts in the patient cohort. Different cell counts (i.e., osteocytes, total number of lacunae and sclerostin-positive osteocytes) were determined in the cortical and trabecular bone of CKD5/transplant patients with different degrees of bone turnover (*n* = 10/group). Data shown as mean ± SD. * *p* ≤ 0.05, ** *p* ≤ 0.01, *** *p* ≤ 0.001.

In both the cortical and trabecular bone, significantly less osteocytes per bone area were detected in patients presenting low bone turnover ROD as compared to the other two groups (Figure 3). The results remained consistent when the number of empty lacunae was also included (indicated as ‘total number of lacunae’). In both the low and normal turnover groups, a higher number of osteocytes was counted in the trabecular bone compartment compared to cortical bone.

In addition, a strong downward trend in the number of sclerostin-positive osteocytes per bone area was noticed in both bone compartments as turnover increases, reaching borderline significance (*p* = 0.051) in trabecular bone. Moreover, when comparing the cortical and trabecular bone compartment, consistently higher numbers of sclerostin-positive osteocytes were counted in cortical bone in all patient groups.

Correlating the cell counts with trabecular histomorphometric bone parameters provided further insights (Table 4). First, the number of osteocytes per bone area is positively correlated with BFR and with B.Ar and osteoid parameters, which are major criteria for ROD classification in histomorphometric analysis for CKD. Second, in line with the BFR, an increased amount of osteocytes turned out to be associated with a more active bone mineralization;, as evidenced by the positive correlations with mineral apposition rate (MAR) and Aj.AR and the reverse association with Mlt. These latter correlations behave in the opposite way when sclerostin-positive osteocytes are considered (Table 4). Compared to the osteocytic cell nuclei, similar results were found when the total number of lacunae was considered (data not shown).

#### 3.2.3. Specific Bone Turnover Parameters Reflect Osteocytic Sclerostin Expression

In Table 5, bone sclerostin expression in both bone compartments is correlated with serum parameters in all patients included in our study. Interestingly, considerable higher sclerostin expression in both cortical and trabecular bone was observed in patients with higher eGFR (thus lower bone turnover groups). iPTH and specific markers of bone turnover—i.e., ALP, BALP, PINP and TRAP5b—were all inversely associated with the number of sclerostin-positive osteocytes per bone area, especially in the cortical bone compartment.

## 4. Discussion

CKD is a serious and complex clinical condition going along with various co-morbidities. Complications can include ROD, a range of bone disorders classified by bone turnover, mineralization and volume [8,10]. Nowadays, quantitative histomorphometric analysis of a bone biopsy is still considered the gold standard for diagnosis of renal bone disease. However, it is invasive and requires particular skills for the surgical intervention and for histomorphometric evaluation. Moreover, the whole process of biopsy, sample preparation and microscopic readout is time consuming. Therefore, there is a continuous search for less invasive and more rapid diagnostic methods, which may also be important for monitoring the progression of CKD. On the other hand, the development and use of additional histological and histomorphometric parameters of a bone biopsy may broaden insight in bone metabolism and lead to the discovery of new circulating biochemical markers that are useful for diagnosis of ROD. Currently, measurement of PTH is still most frequently used. During the last decade, however, the interest in the role of sclerostin in the pathophysiological context of CKD has been growing, in particular its interaction with PTH and their potential reciprocal inverse effect on bone turnover and mineralization [20,33].

**Figure 4:**
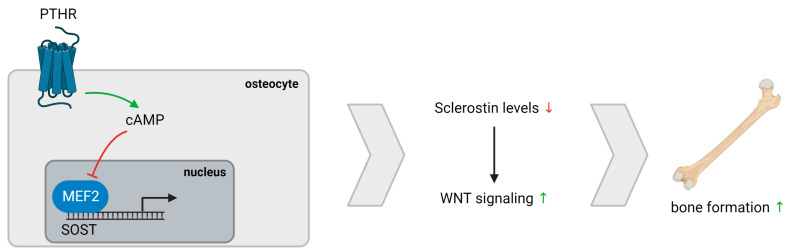
PTH/Sclerostin interaction. PTH exerts a direct effect on cAMP signaling, resulting in reduced SOST mRNA expression due to impairment of the MEF2-stimulated *SOST* promotor. Lower sclerostin levels cause increased WNT signaling and, eventually, bone formation. Abbreviation: PTHR, parathyroid hormone receptor.

We demonstrate an inverse association between serum iPTH levels and sclerostin expression in trabecular bone of PTX rats, which is in line with previous reports suggesting a direct effect on SOST mRNA suppression in bone, most presumably via the cAMP signaling pathway and subsequent inhibition of the MEF2-stimulated *SOST* promotor (Figure 4) [34,35,36,37,38]. Although no differences in both histomorphometric analysis and osteocytic cell counts were observed when comparing PTX and non-PTX rats with normal renal function, a borderline significantly higher number of sclerostin-positive osteocytes was noticed in the trabecular bone of PTX rats. Interestingly, Pires et al. described a similar increase in sclerostin after parathyroidectomy in patients with CKD and secondary hyperparathyroidism [39]. Moreover, osteocytic sclerostin positivity in PTX rats and in our patient cohort was associated with an impaired mineralization, as evidenced by the negative correlation with Aj.AR and positive correlation with Mlt. Hence, our data are in line with previous findings indicating that sclerostin expression inhibits bone mineralization, supposedly by regulating matrix extracellular phosphoglycoprotein (MEPE), another osteocyte-derived factor [33].

Standard histomorphometric analysis of our patient cohort indicates that quantitative analysis of the osteocyte is worth being performed given its role in normal bone metabolism. Our findings of a decreased number of osteocytes per bone area in the low bone turnover group might provide information on the evolution of bone turnover during the natural course of ROD or different interventions over a period of time. Moreover, this group, presenting with the highest number of sclerostin-positive osteocytes per bone area, is in agreement with the known antagonizing effect of sclerostin on bone turnover [40,41]. Taken together, the quantification of osteocytic sclerostin expression, either or not in combination with the assessment of the number of osteocytes per trabecular and/or cortical bone area, may be of value for bone histomorphometric analysis as a histological marker in clinical situations where interventions with the potential to modify bone sclerostin are considered. For our analysis, one must know that the true number of osteocytes was consistently underestimated when only the nuclei were quantified. However, the highly similar results obtained when the total number of lacunae was considered versus only the osteocytic cell nuclei indicate that the cell population counted is representative. Additionally, in both our PTX study and the patient cohort we demonstrated a higher number of osteocytes in the trabecular bone—i.e., the more metabolically active bone compartment—emphasizing the importance of osteocytes in the bone remodeling process [42,43].

Circulating sclerostin has been suggested for the non-invasive diagnosis of ROD. We have previously reported a positive association between bone and serum sclerostin in patients with different stages of CKD [44], which could not be confirmed in the current cohort. This is in line with the loss of correlation between serum and osseous sclerostin in corticosteroid-treated patients in our previous report [44]. Moreover, since it has been reported that serum sclerostin levels highly depend on renal function [25,45], our lack of correlation could, at least to a certain extent, be explained by the fact that eGFR levels in the high turnover group were considerably higher than in low and normal turnover patients. Hence, the expected lower serum sclerostin levels in the high turnover group could be confounded by more pronounced renal retention. The same applies for the relationship between serum iPTH and osseous sclerostin expression, which may be largely driven by altered kidney function. It has also been reported that changes in iPTH occur more quickly than in serum sclerostin, which may, at least in part, explain the different trends in serum iPTH and sclerostin levels [46]. In addition, based on observations in both our rat study and human study, one may suggest that the changes in serum occur later than in bone tissue. This is supported by previous findings showing that serum sclerostin levels start to rise from CKD stage 3, whilst changes in osseous sclerostin expression can be noticed already in early CKD stages [25,47,48,49]. Further evidence for the above statements is provided by the fact that in contrast to serum sclerostin: (i) significant correlations with bone sclerostin levels and (ii) significantly different values between the different turnover groups were found for the other biochemical parameters under study, which do not, or at least to a much lesser extent, depend on renal function. These biochemical factors include BALP (bone turnover/mineralization), PINP (bone formation) and TRAP5b (bone resorption) [50]. Interestingly, these factors correlated inversely with osteocytic sclerostin expression, particularly in the cortical bone compartment. The differences between bone compartments, combined with the consistently higher sclerostin positivity in cortical bone tissue, indicates that both compartments are worth being included for histomorphometric evaluation to better understand the complex pathophysiology of ROD and perhaps other non-CKD related bone diseases, e.g., osteoporosis, where cortical and trabecular pathology can affect fracture risk independently [51]. The most importantlimitation of our study is the large heterogeneity of our patient population with regard to CKD stage and the lack of molecular data. Hence, it would be of interest to continue the investigations in a more homogeneous cohort.

In summary, to the best of our knowledge, this is the first demonstration of differences in associations of skeletal sclerostin expression with altered bone turnover in cortical and trabecular bone in ROD and post-PTX hypoparathyroidism. Sclerostin expression in bone was found relevant in the evaluation of bone turnover and may have potential as therapeutic target in the treatment of impaired mineralization in the clinical setting of ROD, although further investigation is needed. To which extent circulating sclerostin, either alone or in combination with other biomarkers, may serve as a marker for the clinical evaluation of ROD remains to be elucidated in larger cohorts.

## Figures and Tables

**Table 1:** Correlations between bone histomorphometric parameters in trabecular bone and cell counts.

Parameter (Unit)	Osteocytes per Bone Area		Sclerostin-Positive Osteocytes per Bone Area	
	PTX, *n* = 10	Non-PTX, *n* = 9	PTX, *n* = 10	Non-PTX, *n* = 9
BFR (µm^2^/mm^2^/day)	−0.050 (0.891)	0.048 (0.911)	−0.506 (0.135)	−0.238 (0.570)
B.Ar (%)	−0.248 (0.489)	**−0.786 (0.021)**	0.333 (0.347)	0.429 (0.289)
O.Ar (%)	−0.134 (0.713)	−0.143 (0.736)	−0.456 (0.185)	0.262 (0.531)
O.Pm (%)	−0.146 (0.688)	−0.262 (0.531)	−0.468 (0.172)	0.333 (0.420)
O.Wi (µm)	0.061 (0.868)	−0.071 (0.867)	−0.334 (0.345)	0.024 (0.955)
Ob.Pm.O (%)	0.055 (0.880)	**−0.838 (0.009)**	−0.178 (0.623)	0.659 (0.076)
Ob.Pm.T (%)	0.006 (0.987)	−0.647 (0.083)	−0.276 (0.440)	0.443 (0.272)
E.Pm (%)	0.418 (0.229)	0.048 (0.911)	0.382 (0.276)	−0.643 (0.086)
Oc.Pm.E (%)	0.042 (0.907)	−0.143 (0.736)	0.309 (0.385)	−0.095 (0.823)
Oc.Pm.T (%)	0.370 (0.293)	−0.119 (0.779)	0.430 (0.214)	−0.548 (0.160)
MAR (µm/day)	−0.263 (0.464)	−0.405 (0.320)	−0.506 (0.135)	0.071 (0.867)
Aj.AR (µm/day)	−0.163 (0.654)	0.238 (0.570)	**−0.719 (0.019)**	−0.619 (0.102)
Mlt (days)	0.188 (0.604)	−0.048 (0.911)	**0.757 (0.011)**	0.381 (0.352)
Omt (days)	0.388 (0.569)	0.238 (0.570)	0.569 (0.086)	0.167 (0.693)

Abbreviations: BFR, bone formation rate; B.Ar, bone area; O.Ar, osteoid area; O.Pm, osteoid perimeter; O.Wi, osteoid width; Ob.Pm.O, osteoblast perimeter to osteoid perimeter; Ob.Pm.T, osteoid perimeter to total perimeter; E.Pm, eroded perimeter; Oc.Pm.E, osteoclast perimeter to eroded perimeter; Oc.Pm.T, osteoclast perimeter to total perimeter; MAR, mineralization apposition rate; Aj.AR, adjusted apposition rate; Mlt, mineralization lag time; Omt, osteoid maturation time. Data shown as correlation coefficient (*p*-value). Statistically significant values (*p* ≤ 0.05) are shown in bold.

**Table 2:** Correlations between serum ipth and sclerostin expression per bone area.

Compartment	PTX, *n* = 10	Non-PTX, *n* = 9
Cortical	−0.095 (0.823)	0.071 (0.867)
Trabecular	**−0.714 (0.047)**	−0.429 (0.289)

Data shown as correlation coefficient (*p*-value). Statistically significant values (*p* ≤ 0.05) are shown in bold.

**Table 3:** Characteristics of the different patient groups.

Parameter (Unit)	All Patients	Low Turnover	Normal Turnover	High Turnover	*p*-Value
Demographics					
Patients (male)	30 (19)	10 (8)	10 (8)	10 (3)	**0.041**
Age (years)	52.1 (15.7)	58.0 (12.6)	51.2 (6.6)	47.9 (15.0)	0.236
Status (Tx/CKD5)	20/10	10/0	7/3	3/7	**0.0039**
Histomorphometric parameters					
BFR (µm^2^/mm^2^/day)	379.92 (551.3)	54.07 (41.80)	379.92 (151.66)	827.61 (776.26)	**0.000 ^abc^**
B.Ar (%)	19.61 (7.78)	15.50 (3.79)	21.16 (3.68)	23.51 (8.25)	**0.039 ^a^**
O.Ar (%)	4.76 (4.68)	1.44 (3.56)	4.56 (3.16)	7.31 (5.51)	**0.034 ^c^**
O.Pm (%)	37.04 (27.23)	16.26 (21.16)	41.55 (49.06)	46.32 (18.11)	**0.010 ^ac^**
O.Wi (µm)	8.29 (4.25)	6.89 (1.47)	8.25 (3.89)	10.38 (2.29)	**0.016 ^c^**
Ob.Pm.O (%)	25.65 (36.68)	23.41 (29.34)	34.94 (29.00)	28.38 (27.92)	0.440
Ob.Pm.T (%)	7.01 (16.81)	2.45 (4.52)	9.74 (17.88)	12.32 (17.51)	**0.029 ^c^**
E.Pm (%)	3.14 (3.17)	2.15 (2.21)	3.95 (3.87)	3.96 (6.71)	0.076
Oc.Pm.E (%)	31.91 (22.98)	26.93 (32.86)	32.95 (11.44)	25.78 (21.53)	0.715
Oc.Pm.T (%)	0.92 (1.03)	0.48 (0.89)	1.13 (1.08)	0.84 (2.68)	0.120
MAR (µm/day)	0.89 (0.45)	0.64 (0.08)	0.87 (0.15)	1.20 (0.30)	**0.000 ^abc^**
Aj.AR (µm/day)	0.35 (0.36)	0.06 (0.18)	0.39 (0.22)	0.63 (0.17)	**0.000 ^abc^**
Mlt (days)	26.39 (31.47)	94.36 (143.87)	27.68 (13.12)	14.73 (8.97)	**0.001 ^ac^**
Omt (days)	9.71 (3.58)	10.70 (1.87)	9.89 (4.42)	8.90 (2.80)	0.246
Serum factors					
Creatinine (mg/dL)	1.71 (4.52)	1.47 (0.37)	1.81 (1.05)	6.44 (4.84)	0.053
eGFR	41 (42)	49 (17)	42 (22)	7 (17)	**0.012 ^c^**
(mL/min/1.73 m^2^)					
Calcium (mmol/L)	2.43 (0.20)	2.43 (0.22)	2.42 (0.22)	2.43 (0.26)	0.820
Phosphate (mmol/L)	1.05 (0.55)	0.97 (0.28)	0.97 (0.31)	1.48 (0.68)	0.197
25-OH Vit D (µg/L)	33.50 (14.63)	35.05 (9.58)	33.20 (8.70)	31.00 (13.30)	0.595
iPTH (ng/dL)	109.20 (365.50)	66.35 (45.33)	111.60 (54.10)	427.90 (576.90)	**0.005 ^c^**
ALP (U/L)	88.12 (73.45)	74.5 (30.78)	92.00 (37.00)	174.00 (97.06)	**0.001 ^bc^**
BALP (µg/L)	23.36 (16.53)	15.61 (11.04)	27.68 (12.50)	47.52 (12.33)	**0.007 ^c^**
PINP (ng/mL)	80.06 (83.89)	46.68 (38.15)	88.33 (70.13)	142.28 (17.31)	**0.002 ^c^**
TRAP5b (U/L)	4.30 (3.38)	3.60 (1.56)	3.99 (3.12)	7.86 (1.83)	**0.005 ^c^**
Sclerostin (ng/mL)	0.82 (0.36)	0.82 (0.26)	0.84 (0.41)	0.86 (0.52)	0.859

Abbreviations: Tx, transplantation; CKD5, chronic kidney disease stage 5; BFR, bone formation rate; B.Ar, bone area; O.Ar, osteoid area; O.Pm, osteoid perimeter; O.Wi, osteoid width; Ob.Pm.O, osteoblast perimeter to osteoid perimeter; Ob.Pm.T, osteoid perimeter to total perimeter; E.Pm, eroded perimeter; Oc.Pm.E, osteoclast perimeter to eroded perimeter; Oc.Pm.T, osteoclast perimeter to total perimeter; MAR, mineralization apposition rate; Aj.AR, adjusted apposition rate; Mlt, mineralization lag time; Omt, osteoid maturation time. eGFR, estimated glomerular filtration rate; iPTH, intact parathyroid hormone; ALP, total alkaline phosphatase; BALP, bone alkaline phosphatase; PINP, *N*-terminal propeptide of type I procollagen; TRAP5b, tartrate-resistant acid phosphatase 5b. Data shown as median (IQR). Statistically significant results (*p* < 0.05, Bonferroni corrected) are shown in bold. Significant differences are shown between the ^a^ low and normal bone turnover groups, ^b^ normal and high turnover groups or ^c^ low and high turnover groups.

**Table 4:** Correlations between bone histomorphometric parameters in trabecular bone and cell counts.

Parameter (Unit)	Osteocytes per Bone Area (*p*-Value)	Sclerostin Expression per Bone Area (*p*-Value)
BFR (µm^2^/mm^2^/day)	**0.694 (0.000)**	−0.339 (0.072)
B.Ar (%)	0.237 (0.215)	−0.110 (0.569)
O.Ar (%)	**0.509 (0.005)**	0.009 (0.962)
O.Pm (%)	**0.542 (0.002)**	−0.003 (0.988)
O.Wi (µm)	**0.397 (0.033)**	0.000 (0.998)
Ob.Pm.O (%)	−0.046 (0.814)	0.100 (0.605)
Ob.Pm.T (%)	0.223 (0.263)	−0.005 (0.980)
E.Pm (%)	0.319 (0.091)	−0.251 (0.189)
Oc.Pm.E (%)	0.218 (0.256)	−0.246 (0.199)
Oc.Pm.T (%)	0.259 (0.192)	−0.325 (0.098)
MAR (µm/day)	**0.494 (0.006)**	−0.326 (0.085)
Aj.AR (µm/day)	**0.590 (0.001)**	**−0.481 (0.008)**
Mlt (days)	**−0.469 (0.010)**	**0.526 (0.003)**
Omt (days)	−0.149 (0.440)	0.287 (0.132)

Abbreviations: BFR, bone formation rate; B.Ar, bone area; O.Ar, osteoid area; O.Pm, osteoid perimeter; O.Wi, osteoid width; Ob.Pm.O, osteoblast perimeter to osteoid perimeter; Ob.Pm.T, osteoid perimeter to total perimeter; E.Pm, eroded perimeter; Oc.Pm.E, osteoclast perimeter to eroded perimeter; Oc.Pm.T, osteoclast perimeter to total perimeter; MAR, mineralization apposition rate; Aj.AR, adjusted apposition rate; Mlt, mineralization lag time; Omt, osteoid maturation time. Data shown as correlation coefficient (*p*-value). Statistically significant values (*p* ≤ 0.05) are shown in bold.

**Table 5:** Bone sclerostin expression, serum biochemistry and circulating markers of bone turnover.

Parameter (Unit)	Sclerostin Expression Cortical Bone (*p*-Value)	Sclerostin Expression Trabecular Bone (*p*-Value)
Age (years)	0.242 (0.198)	0.084 (0.664)
Creatinine (mg/dL)	**−0.422 (0.025)**	−0.346 (0.077)
eGFR (mL/min/1.73 m^2^)	**0.414 (0.028)**	**0.383 (0.049)**
Calcium (mmol/L)	0.056 (0.775)	0.054 (0.791)
Phosphate (mmol/L)	−0.272 (0.162)	−0.195 (0.329)
25-OH Vit D (µg/L)	−0.235 (0.230)	−0.201 (0.316)
iPTH (ng/dL)	**−0.561 (0.002)**	−0.363 (0.057)
ALP (U/L)	**−0.387 (0.042)**	−0.340 (0.083)
BALP (µg/L)	**−0.528 (0.012)**	−0.293 (0.186)
PINP (ng/mL)	**−0.541 (0.006)**	**−0.474 (0.019)**
TRAP5b (U/L)	**−0.562 (0.003)**	−0.234 (0.261)
Sclerostin (ng/dL)	−0.250 (0.219)	−0.136 (0.508)

eGFR, estimated glomerular filtration rate; iPTH, parathyroid hormone; ALP, total alkaline phosphatase; BALP, bone alkaline phosphatase; PINP, *N*-terminal propeptide of type I procollagen; TRAP5b, tartrate-resistant acid phosphatase 5b. Data shown as correlation coefficient (*p*-value). Statistically significant results (*p* ≤ 0.05) are shown in bold.

## Data Availability

The data presented in this study are all available in the article and the supplementary materials. No other data were created or analyzed.

## Supplementary Materials

The following supporting information can be downloaded at: https://www.mdpi.com/article/10.3390/nu15030598/s1, Table S1: Bone Histomorphometric Parameters. Table S2: Histomorphometric Parameters of the (Non-)PTX Rats.
