# Supplementary material for: Osteocytic Sclerostin Expression as an Indicator of Altered Bone Turnover
Source: Nutrients. 2023 Jan 23;15(3):598. doi: 10.3390/nu15030598 (PMC9921466; doi:10.3390/nu15030598)
Supplement: Supplementary file 1 [file nutrients-15-00598-s001.zip › nutrients-2144302-supplementary.pdf]

Table S1. BONE HISTOMORPHOMETRIC PARAMETERS.

Histomorphometric parameter (abbreviation, unit)	Definition of the histomorphometric parameter
**Static parameters**	
Bone area (B.Ar, %)	Percentage of tissue area, consisting of trabecular bone tissue (mineralized bone and osteoid)
Osteoid area (O.Ar, %)	Percentage of bone area consisting of osteoid
Osteoid perimerer (O.Pm, %)	Percentage of total bone perimeter covered by osteoid
Osteoid width (O.Wi, μm)	Average width of the osteoid seams
Osteoblast perimeter to osteoid perimeter (Ob.Pm.O, %)	Percentage of the osteoid perimeter covered by active osteoblasts
Osteoblast perimeter to total perimeter (Ob.Pm.T, %)	Percentage of the total bone perimeter covered by active osteoblasts
Eroded perimeter (E.Pm, %)	Percentage of the total bone perimeter that has been eroded by osteoclasts
Osteoclast perimeter to eroded perimeter (Oc.Pm.E, %)	Percentage of the eroded perimeter covered by resorbing osteoclasts
Osteoclast perimeter to total perimeter (Oc.Pm.T, %)	Percentage of the total bone perimeter covered by resorbing osteoclasts
**Dynamic parameters**	
Bone formation rate (BFR, $\mu m^2/mm^2$/day)	Area of bone, formed per unit of time, and existing bone
Mineral apposition rate (MAR, μm/day)	The rate by which osteoid is mineralized (average distance between the midpoints of two tetracycline labels, divided by the time interval between the administration of these labels)
Adjusted apposition rate (Aj.AR, μm/day)	The mineral apposition rate, averaged over the entire osteoid surface
Mineralization lag time (Mlt, days)	Time interval between the deposition of the osteoid and its subsequent mineralization, averaged over the entire life span of the osteoid seam
Osteoid maturation time (Omt, days)	Time interval between the onset of matrix deposition and the onset of mineralization at each bone-forming site

Based on standardized nomenclature, symbols and units for bone histomorphometry, as published by Dempster et al. [32].

Table S2. HISTOMORPHOMETRIC PARAMETERS OF THE (NON-)PTX RATS

**Parameter (unit)**	**PTX, *N=10***	**non-PTX, *N=9***	**p-value**
BFR (µm²/mm²/day)	183.62 (253.16)	153.84 (64.80)	0.491
B.Ar (%)	0.28 (0.072)	0.27 (0.069)	1.000
O.Ar (%)	0.0031 (0.0059)	0.0031 (0.0034)	0.321
O.Pm (%)	0.028 (0.064)	0.040 (0.044)	0.481
O.Wi (µm)	3.01 (0.55)	2.74 (0.25)	0.370
Ob.Pm.O (%)	0.30 (0.19)	0.27 (0.10)	0.423
Ob.Pm.T (%)	0.0087 (0.032)	0.011 (0.013)	0.383
E.Pm (%)	0.015 (0.010)	0.0062 (0.0064)	0.805
Oc.Pm.E (%)	0.27 (0.079)	0.21 (0.047)	0.093
Oc.Pm.T (%)	0.0041 (0.0026)	0.0014 (0.0014)	0.200
MAR (µm/day)	2.29 (0.62)	1.77 (0.70)	0.282
Aj.AR (µm/day)	0.90 (0.56)	0.89 (0.79)	1.000
Mlt (days)	3.17 (6.49)	3.17 (4.64)	1.000
Omt (days)	1.25 (0.40)	1.49 (0.52)	0.573

Abbreviations: see Table A1. Data shown as median (IQR).
